# *N*-(2,5-Dimethyl­phen­yl)-4-methyl­benzene­sulfonamide

**DOI:** 10.1107/S1600536809051174

**Published:** 2009-12-04

**Authors:** B. Thimme Gowda, Sabine Foro, P. G. Nirmala, Hartmut Fuess

**Affiliations:** a Department of Chemistry, Mangalore University, Mangalagangotri 574 199, Mangalore, India; b Institute of Materials Science, Darmstadt University of Technology, Petersenstrasse 23, D-64287 Darmstadt, Germany

## Abstract

In the crystal structure of the title compound, C_15_H_17_NO_2_S, the conformation of the N—C bond in the C—SO_2_—NH—C segment has *gauche* torsions with respect to the S=O bonds. The mol­ecule is bent at the S atom with a C—SO_2_—NH—C torsion angle of −61.0 (2)°. The dihedral angle between the two aromatic rings is 49.4 (1)°. The crystal structure features inversion-related dimers linked by pairs of N—H⋯O hydrogen bonds.

## Related literature

For our study of the effects of substituents on the structures of *N*-(ar­yl)-aryl­sulfonamides, see: Gowda *et al.* (2009**a* ▶,b* ▶). For related structures, see: Gelbrich *et al.* (2007 ▶); Perlovich *et al.* (2006 ▶)
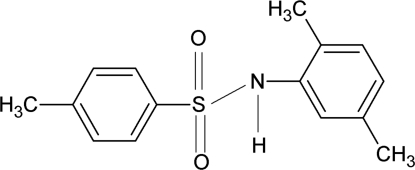


## Experimental

### 

#### Crystal data

C_15_H_17_NO_2_S
*M* *_r_* = 275.36
Triclinic, 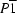

*a* = 8.6397 (7) Å
*b* = 9.7067 (8) Å
*c* = 10.518 (1) Å
α = 66.97 (1)°
β = 81.37 (1)°
γ = 64.82 (1)°
*V* = 734.47 (11) Å^3^
*Z* = 2
Cu *K*α radiation
μ = 1.94 mm^−1^
*T* = 299 K
0.50 × 0.30 × 0.08 mm

#### Data collection

Enraf–Nonius CAD-4 diffractometer
Absorption correction: ψ scans (North *et al.*, 1968 ▶) *T*_min_ = 0.444, *T*_max_ = 0.861
3926 measured reflections
2603 independent reflections
2324 reflections with *I* > 2σ(*I*)
*R*_int_ = 0.040

#### Refinement

*R*[*F* ^2^ > 2σ(*F* ^2^)] = 0.047
*wR*(*F* ^2^) = 0.140
*S* = 1.18
2603 reflections
176 parameters
H-atom parameters constrained
Δρ_max_ = 0.39 e Å^−3^
Δρ_min_ = −0.46 e Å^−3^

### 

Data collection: *CAD-4-PC* (Enraf–Nonius, 1996 ▶); cell refinement: *CAD-4-PC*; data reduction: *REDU4* (Stoe & Cie, 1987 ▶); program(s) used to solve structure: *SHELXS97* (Sheldrick, 2008 ▶); program(s) used to refine structure: *SHELXL97* (Sheldrick, 2008 ▶); molecular graphics: *PLATON* (Spek, 2009 ▶); software used to prepare material for publication: *SHELXL97*.

## Supplementary Material

Crystal structure: contains datablocks I, global. DOI: 10.1107/S1600536809051174/ng2695sup1.cif

Structure factors: contains datablocks I. DOI: 10.1107/S1600536809051174/ng2695Isup2.hkl

Additional supplementary materials:  crystallographic information; 3D view; checkCIF report

## Figures and Tables

**Table 1:** Hydrogen-bond geometry (Å, °)

*D*—H⋯*A*	*D*—H	H⋯*A*	*D*⋯*A*	*D*—H⋯*A*
N1—H1*N*⋯O1^i^	0.86	2.28	2.957 (2)	135

Symmetry code: (i) 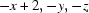.

**Fig. 1.**
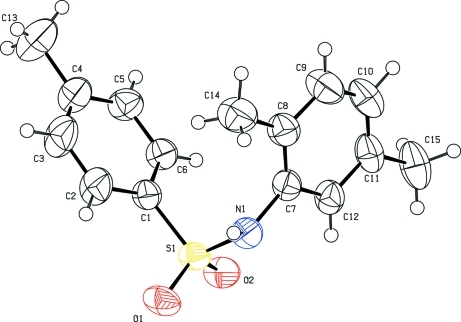
Molecular structure of (I), showing the atom labelling scheme and displacement ellipsoids are drawn at the 50% probability level.

**Fig. 2.**
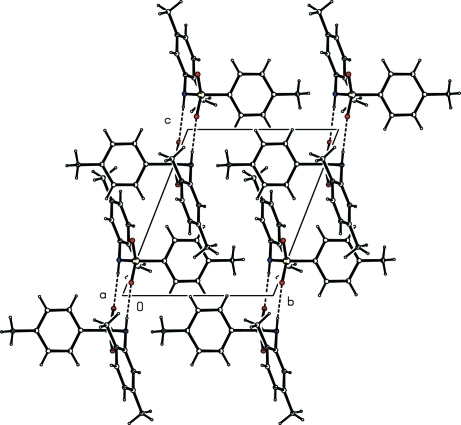
Molecular packing of (I) with hydrogen bonding shown as dashed lines.

## supplementary crystallographic information

### Comment

As part of a study of the substituent effects on the crystal structures of *N*-(aryl)-arylsulfonamides (Gowda *et al.*, 2009*a,b*), in the present work, the structure of 4-methyl-*N*-(2,5-dimethylphenyl)benzenesulfonamide (I) has been determined. The conformation of the N—C bond in the C—SO_2_—NH—C segment of the structure has *gauche* torsions with respect to the S═O bonds (Fig. 1). The molecule is bent at the S atom with the C—SO_2_—NH—C torsion angle of -61.0 (2)°, compared to the values of -61.8 (2)° in 4-methyl-*N*-(3,4-dimethylphenyl)benzenesulfonamide (II), -51.6 (3)° in 4-Methyl-*N*-(phenyl)benzenesulfonamide (III) (Gowda *et al.*, 2009*b*) and 62.7 (2)° in *N*-(2,5-dimethylphenyl)benzenesulfonamide (IV) (Gowda *et al.*, 2009*a*). The two benzene rings in (I) are tilted relative to each other by 49.4 (1)°, compared to the values of 47.8 (1)° in (II), 68.4 (1)° in (III) and 40.4 (1)° in (IV). The other bond parameters in (I) are similar to those observed in (II), (III), (IV) and other aryl sulfonamides (Perlovich *et al.*, 2006; Gelbrich *et al.*, 2007). The pairs of N—H···O hydrogen bonds (Table 1) pack the molecules into infinite chains parallel to the *c*-axis (Fig. 2).

### Experimental

The solution of toluene (10 cc) in chloroform (40 cc) was treated dropwise with chlorosulfonic acid (25 cc) at 0 ° C. After the initial evolution of hydrogen chloride subsided, the reaction mixture was brought to room temperature and poured into crushed ice in a beaker. The chloroform layer was separated, washed with cold water and allowed to evaporate slowly. The residual 4-methylbenzenesulfonylchloride was treated with 2,5-dimethylaniline in the stoichiometric ratio and boiled for ten minutes. The reaction mixture was then cooled to room temperature and added to ice cold water (100 cc). The resultant 4-methyl-*N*-(2,5-dimethylphenyl)benzenesulfonamide was filtered under suction and washed thoroughly with cold water. It was then recrystallized to constant melting point from dilute ethanol. The purity of the compound was checked and characterized by recording its infrared and NMR spectra. The single crystals used in X-ray diffraction studies were grown in ethanolic solution by a slow evaporation at room temperature.

### Refinement

The H atoms were positioned with idealized geometry using a riding model [N—H = 0.86 Å, C—H = 0.93–0.96 Å] and were refined with isotropic displacement parameters (set to 1.2 times of the *U*_eq_ of the parent atom).

### Crystal data

**Table:**

C_15_H_17_NO_2_S	*Z* = 2
*M**_r_* = 275.36	*F*(000) = 292
Triclinic, *P*1	*D*_x_ = 1.245 Mg m^−^^3^
Hall symbol: -P 1	Cu *K*α radiation, λ = 1.54180 Å
*a* = 8.6397 (7) Å	Cell parameters from 25 reflections
*b* = 9.7067 (8) Å	θ = 7.2–23.8°
*c* = 10.518 (1) Å	µ = 1.94 mm^−^^1^
α = 66.97 (1)°	*T* = 299 K
β = 81.37 (1)°	Prism, colourless
γ = 64.82 (1)°	0.50 × 0.30 × 0.08 mm
*V* = 734.47 (11) Å^3^	

### Data collection

**Table:**

Enraf–Nonius CAD-4 diffractometer	2324 reflections with *I* > 2σ(*I*)
Radiation source: fine-focus sealed tube	*R*_int_ = 0.040
graphite	θ_max_ = 66.9°, θ_min_ = 4.6°
ω/2θ scans	*h* = −10→4
Absorption correction: ψ scan (North *et al.*, 1968)	*k* = −11→11
*T*_min_ = 0.444, *T*_max_ = 0.861	*l* = −12→12
3926 measured reflections	3 standard reflections every 120 min
2603 independent reflections	intensity decay: 1.0%

### Refinement

**Table:**

Refinement on *F*^2^	Secondary atom site location: difference Fourier map
Least-squares matrix: full	Hydrogen site location: inferred from neighbouring sites
*R*[*F*^2^ > 2σ(*F*^2^)] = 0.047	H-atom parameters constrained
*wR*(*F*^2^) = 0.140	*w* = 1/[σ^2^(*F*_o_^2^) + (0.0683*P*)^2^ + 0.2017*P*] where *P* = (*F*_o_^2^ + 2*F*_c_^2^)/3
*S* = 1.18	(Δ/σ)_max_ = 0.001
2603 reflections	Δρ_max_ = 0.39 e Å^−^^3^
176 parameters	Δρ_min_ = −0.46 e Å^−^^3^
0 restraints	Extinction correction: *SHELXL97* (Sheldrick, 2008), Fc^*^=kFc[1+0.001xFc^2^λ^3^/sin(2θ)]^-1/4^
Primary atom site location: structure-invariant direct methods	Extinction coefficient: 0.0133 (16)

### Special details

**Table:**

Geometry. All e.s.d.'s (except the e.s.d. in the dihedral angle between two l.s. planes) are estimated using the full covariance matrix. The cell e.s.d.'s are taken into account individually in the estimation of e.s.d.'s in distances, angles and torsion angles; correlations between e.s.d.'s in cell parameters are only used when they are defined by crystal symmetry. An approximate (isotropic) treatment of cell e.s.d.'s is used for estimating e.s.d.'s involving l.s. planes.
Refinement. Refinement of *F*^2^ against ALL reflections. The weighted *R*-factor *wR* and goodness of fit *S* are based on *F*^2^, conventional *R*-factors *R* are based on *F*, with *F* set to zero for negative *F*^2^. The threshold expression of *F*^2^ > σ(*F*^2^) is used only for calculating *R*-factors(gt) *etc*. and is not relevant to the choice of reflections for refinement. *R*-factors based on *F*^2^ are statistically about twice as large as those based on *F*, and *R*- factors based on ALL data will be even larger.

### Fractional atomic coordinates and isotropic or equivalent isotropic displacement parameters (Å^2^)

**Table:**

	*x*	*y*	*z*	*U*_iso_*/*U*_eq_	
S1	1.05078 (6)	−0.00156 (6)	0.20853 (5)	0.0456 (2)	
O1	1.1338 (2)	0.0247 (2)	0.07776 (16)	0.0591 (5)	
O2	1.1518 (2)	−0.0780 (2)	0.33191 (16)	0.0560 (4)	
N1	0.9516 (2)	−0.1130 (2)	0.21337 (18)	0.0487 (5)	
H1N	0.9621	−0.1456	0.1462	0.058*	
C1	0.8925 (3)	0.1869 (3)	0.2088 (2)	0.0451 (5)	
C2	0.8295 (4)	0.3162 (3)	0.0855 (3)	0.0646 (7)	
H2	0.8739	0.3063	0.0018	0.078*	
C3	0.6998 (4)	0.4602 (3)	0.0887 (3)	0.0761 (8)	
H3	0.6568	0.5471	0.0058	0.091*	
C4	0.6323 (4)	0.4792 (3)	0.2106 (3)	0.0651 (7)	
C5	0.6989 (3)	0.3478 (3)	0.3329 (3)	0.0574 (6)	
H5	0.6554	0.3583	0.4166	0.069*	
C6	0.8281 (3)	0.2021 (3)	0.3331 (2)	0.0494 (5)	
H6	0.8713	0.1151	0.4159	0.059*	
C7	0.8463 (3)	−0.1581 (3)	0.3284 (2)	0.0464 (5)	
C8	0.6706 (3)	−0.0938 (3)	0.3112 (3)	0.0565 (6)	
C9	0.5788 (4)	−0.1463 (4)	0.4267 (3)	0.0734 (8)	
H9	0.4606	−0.1073	0.4188	0.088*	
C10	0.6579 (4)	−0.2540 (4)	0.5520 (3)	0.0726 (8)	
H10	0.5918	−0.2850	0.6270	0.087*	
C11	0.8321 (4)	−0.3172 (3)	0.5696 (2)	0.0591 (6)	
C12	0.9274 (3)	−0.2698 (3)	0.4553 (2)	0.0530 (6)	
H12	1.0459	−0.3126	0.4633	0.064*	
C13	0.4892 (5)	0.6354 (4)	0.2132 (4)	0.0963 (11)	
H13A	0.4227	0.6898	0.1290	0.116*	
H13B	0.5359	0.7052	0.2218	0.116*	
H13C	0.4175	0.6112	0.2903	0.116*	
C14	0.5794 (4)	0.0286 (4)	0.1766 (3)	0.0760 (8)	
H14A	0.6140	−0.0205	0.1080	0.091*	
H14B	0.6079	0.1218	0.1472	0.091*	
H14C	0.4581	0.0628	0.1887	0.091*	
C15	0.9181 (5)	−0.4357 (4)	0.7079 (3)	0.0797 (9)	
H15A	0.8746	−0.5195	0.7457	0.096*	
H15B	0.8949	−0.3784	0.7697	0.096*	
H15C	1.0393	−0.4847	0.6962	0.096*	

### Atomic displacement parameters (Å^2^)

**Table:**

	*U*^11^	*U*^22^	*U*^33^	*U*^12^	*U*^13^	*U*^23^
S1	0.0435 (3)	0.0545 (3)	0.0279 (3)	−0.0142 (2)	0.0042 (2)	−0.0118 (2)
O1	0.0546 (9)	0.0812 (11)	0.0335 (9)	−0.0253 (9)	0.0123 (7)	−0.0194 (8)
O2	0.0487 (8)	0.0694 (10)	0.0350 (8)	−0.0145 (8)	−0.0046 (6)	−0.0129 (7)
N1	0.0570 (10)	0.0528 (10)	0.0319 (9)	−0.0178 (9)	0.0057 (8)	−0.0176 (8)
C1	0.0474 (11)	0.0474 (11)	0.0330 (11)	−0.0172 (9)	0.0009 (8)	−0.0095 (9)
C2	0.0807 (17)	0.0568 (14)	0.0341 (12)	−0.0163 (13)	−0.0006 (11)	−0.0066 (10)
C3	0.094 (2)	0.0539 (14)	0.0496 (16)	−0.0122 (14)	−0.0125 (14)	−0.0029 (12)
C4	0.0660 (15)	0.0511 (13)	0.0680 (18)	−0.0148 (12)	−0.0039 (13)	−0.0195 (12)
C5	0.0587 (14)	0.0623 (14)	0.0497 (14)	−0.0194 (12)	0.0053 (11)	−0.0258 (11)
C6	0.0531 (12)	0.0530 (12)	0.0331 (11)	−0.0165 (10)	0.0011 (9)	−0.0124 (9)
C7	0.0575 (12)	0.0447 (11)	0.0351 (11)	−0.0198 (10)	0.0058 (9)	−0.0153 (9)
C8	0.0596 (14)	0.0594 (13)	0.0472 (14)	−0.0244 (11)	0.0038 (11)	−0.0168 (11)
C9	0.0642 (16)	0.090 (2)	0.0663 (19)	−0.0395 (16)	0.0140 (14)	−0.0240 (16)
C10	0.087 (2)	0.088 (2)	0.0515 (16)	−0.0551 (17)	0.0204 (14)	−0.0198 (14)
C11	0.0856 (18)	0.0582 (14)	0.0406 (13)	−0.0417 (13)	0.0075 (12)	−0.0133 (11)
C12	0.0652 (14)	0.0490 (12)	0.0391 (12)	−0.0226 (11)	0.0016 (10)	−0.0112 (10)
C13	0.097 (2)	0.0616 (17)	0.103 (3)	−0.0021 (17)	−0.011 (2)	−0.0308 (18)
C14	0.0570 (15)	0.088 (2)	0.0616 (17)	−0.0218 (14)	−0.0052 (13)	−0.0117 (15)
C15	0.122 (3)	0.0820 (19)	0.0410 (15)	−0.063 (2)	−0.0003 (15)	−0.0031 (13)

### Geometric parameters (Å, °)

**Table:**

S1—O2	1.4256 (16)	C8—C9	1.391 (4)
S1—O1	1.4341 (15)	C8—C14	1.503 (4)
S1—N1	1.625 (2)	C9—C10	1.371 (4)
S1—C1	1.758 (2)	C9—H9	0.9300
N1—C7	1.442 (3)	C10—C11	1.373 (4)
N1—H1N	0.8600	C10—H10	0.9300
C1—C6	1.379 (3)	C11—C12	1.388 (3)
C1—C2	1.382 (3)	C11—C15	1.510 (4)
C2—C3	1.379 (4)	C12—H12	0.9300
C2—H2	0.9300	C13—H13A	0.9600
C3—C4	1.374 (4)	C13—H13B	0.9600
C3—H3	0.9300	C13—H13C	0.9600
C4—C5	1.388 (4)	C14—H14A	0.9600
C4—C13	1.502 (4)	C14—H14B	0.9600
C5—C6	1.380 (3)	C14—H14C	0.9600
C5—H5	0.9300	C15—H15A	0.9600
C6—H6	0.9300	C15—H15B	0.9600
C7—C8	1.384 (3)	C15—H15C	0.9600
C7—C12	1.396 (3)		
			
O2—S1—O1	118.99 (10)	C9—C8—C14	120.5 (2)
O2—S1—N1	108.50 (10)	C10—C9—C8	121.8 (3)
O1—S1—N1	105.30 (10)	C10—C9—H9	119.1
O2—S1—C1	107.99 (10)	C8—C9—H9	119.1
O1—S1—C1	108.74 (10)	C9—C10—C11	121.7 (2)
N1—S1—C1	106.69 (10)	C9—C10—H10	119.1
C7—N1—S1	121.11 (15)	C11—C10—H10	119.1
C7—N1—H1N	119.4	C10—C11—C12	117.7 (2)
S1—N1—H1N	119.4	C10—C11—C15	121.4 (3)
C6—C1—C2	120.6 (2)	C12—C11—C15	120.9 (3)
C6—C1—S1	119.18 (17)	C11—C12—C7	120.4 (2)
C2—C1—S1	120.17 (18)	C11—C12—H12	119.8
C3—C2—C1	118.9 (2)	C7—C12—H12	119.8
C3—C2—H2	120.6	C4—C13—H13A	109.5
C1—C2—H2	120.6	C4—C13—H13B	109.5
C4—C3—C2	122.0 (2)	H13A—C13—H13B	109.5
C4—C3—H3	119.0	C4—C13—H13C	109.5
C2—C3—H3	119.0	H13A—C13—H13C	109.5
C3—C4—C5	117.9 (2)	H13B—C13—H13C	109.5
C3—C4—C13	121.7 (3)	C8—C14—H14A	109.5
C5—C4—C13	120.4 (3)	C8—C14—H14B	109.5
C6—C5—C4	121.5 (2)	H14A—C14—H14B	109.5
C6—C5—H5	119.3	C8—C14—H14C	109.5
C4—C5—H5	119.3	H14A—C14—H14C	109.5
C1—C6—C5	119.2 (2)	H14B—C14—H14C	109.5
C1—C6—H6	120.4	C11—C15—H15A	109.5
C5—C6—H6	120.4	C11—C15—H15B	109.5
C8—C7—C12	121.7 (2)	H15A—C15—H15B	109.5
C8—C7—N1	120.3 (2)	C11—C15—H15C	109.5
C12—C7—N1	118.0 (2)	H15A—C15—H15C	109.5
C7—C8—C9	116.6 (2)	H15B—C15—H15C	109.5
C7—C8—C14	122.9 (2)		
			
O2—S1—N1—C7	54.45 (18)	S1—C1—C6—C5	−177.31 (18)
O1—S1—N1—C7	−177.12 (16)	C4—C5—C6—C1	0.1 (4)
C1—S1—N1—C7	−61.67 (18)	S1—N1—C7—C8	111.3 (2)
O2—S1—C1—C6	−31.9 (2)	S1—N1—C7—C12	−69.9 (2)
O1—S1—C1—C6	−162.32 (18)	C12—C7—C8—C9	−0.1 (4)
N1—S1—C1—C6	84.6 (2)	N1—C7—C8—C9	178.7 (2)
O2—S1—C1—C2	150.3 (2)	C12—C7—C8—C14	178.9 (3)
O1—S1—C1—C2	19.9 (2)	N1—C7—C8—C14	−2.3 (4)
N1—S1—C1—C2	−93.2 (2)	C7—C8—C9—C10	1.1 (4)
C6—C1—C2—C3	−0.7 (4)	C14—C8—C9—C10	−177.9 (3)
S1—C1—C2—C3	177.0 (2)	C8—C9—C10—C11	−0.7 (5)
C1—C2—C3—C4	0.4 (5)	C9—C10—C11—C12	−0.7 (4)
C2—C3—C4—C5	0.1 (5)	C9—C10—C11—C15	−180.0 (3)
C2—C3—C4—C13	−179.0 (3)	C10—C11—C12—C7	1.7 (4)
C3—C4—C5—C6	−0.4 (4)	C15—C11—C12—C7	−179.0 (2)
C13—C4—C5—C6	178.8 (3)	C8—C7—C12—C11	−1.3 (4)
C2—C1—C6—C5	0.5 (4)	N1—C7—C12—C11	179.9 (2)

### Hydrogen-bond geometry (Å, °)

**Table:**

*D*—H···*A*	*D*—H	H···*A*	*D*···*A*	*D*—H···*A*
N1—H1N···O1^i^	0.86	2.28	2.957 (2)	135

Symmetry codes: (i) −*x*+2, −*y*, −*z*.
